# Reporting new cases of anaemia in primary care settings in Crete, Greece: a rural practice study

**DOI:** 10.1186/1447-056X-11-4

**Published:** 2012-04-25

**Authors:** Christos Lionis, Emmanouil K Symvoulakis, George Duijker, Foteini Anastasiou, Stilianos Dimitrakopoulos, Christina Kladou, Evanggelia Ladoukaki, Kornilia Makri, Chrisoula Petraki, Nektarios Sivaropoulos, Spiridon Sasarolis, Anastasia Stefanaki, Aggeliki Vasilaki, Theodoros Vasilopoulos

**Affiliations:** 1 Clinic of Social and Family Medicine, School of Medicine, University of Crete, Heraklion, Crete, Greece; 2 Research-based network of General Practice in Crete, Heraklion, Greece; 3 Spili Primary Health Care Centre, Spili, Rethymno, Crete, Greece; 4 Charakas Primary Health Care Centre, Charakas, Heraklion, Crete, Greece

## Abstract

**Background:**

Early diagnosis of anaemia represents an important task within primary care settings. This study reports on the frequency of new cases of anaemia among patients attending rural primary care settings in Crete (Greece) and to offer an estimate of iron deficiency anaemia (IDA) frequency in this study group.

**Methods:**

All patients attending the rural primary health care units of twelve general practitioners (GPs) on the island of Crete for ten consecutive working days were eligible to participate in this study. Hemoglobin (Hb) levels were measured by portable analyzers. Laboratory tests to confirm new cases of anaemia were performed at the University General Hospital of Heraklion.

**Results:**

One hundred and thirteen out of 541 recruited patients had a low value of Hb according to the initial measurement obtained by the use of the portable analyzer. Forty five (45.5%) of the 99 subjects who underwent laboratory testing had confirmed anaemia. The mean value of the Hb levels in the group with confirmed anaemia, as detected by the portable analyzer was 11.1 g/dl (95% Confidence Interval (CI) from 10.9 to 11.4) and the respective mean value of the Hb levels obtained from the full blood count was 11.4 g/dl (95% CI from 11.2 to 11.7) (*P *= 0.01). Sixteen out of those 45 patients with anaemia (35.6%) had IDA, with ferritin levels lower than 30 ng/ml.

**Conclusion:**

Keeping in mind that this paper does not deal with specificity or sensitivity figures, it is suggested that in rural and remote settings anaemia is still invisible and point of care testing may have a place to identify it.

## Background

Anaemia is a common haematologic disorder which increasingly occurs in older age groups and its onset is usually insidious and slow [1]. As anemia worsens and due to insufficient circulatory adaptive mechanisms in the elderly, discomfort and functional impairment may be progressively installed [1]. Approximately, 11.0% of men and 10.2% of women ≥ 65 years are anaemic. One third of the elderly patients with anaemia present nutrient deficiencies, conditions which should be readily treated and cautiously linked with possible underlying disorders [2].

Symptoms reported in general practice/family medicine (GP/FM) play an important role in diagnosing anaemia. Fatigue, palpitations, dyspnoea on exertion, and poor concentration are some of the general symptoms related to anaemia, however they can be similarly linked with cancer, thyroid disorders, angina pectoris, and depression respectively [3-5]. Clinical signs like cheilitis, hair loss, fragility of nails and even cognitive impairment in many cases have been commonly associated with anaemia [1,6-8]. Unexplained weight loss, insisting bony pain and other atypical signs deserve to be assessed so as to rule out malignancy or inflammation causes [1]. Diagnosis of anaemia in primary care settings deserves attention and it is considered as essential task for GPs since it is frequently associated with chronic illness. The most common cause of iron deficiency anaemia (IDA) among those aged 50 and above is an occult loss of blood in the gastrointestinal (GI) tract [9]. Premenopausal women also have a high prevalence of IDA not only secondary to menstrual blood loss but also due to low intake of dietary iron [10]. Inadequate absorption of iron is another well established cause of IDA [11]. The presence of IDA can also be the first sign of GI disorders including inflammatory bowel diseases, celiac disease and atrophic gastritis [12,13]. Other known etiologies of IDA are intense physical exercise, chronic diseases which are paired with inflammatory processes, and the intake of agents that can subsequently interfere with the absorption of iron. In general, unless there is a clinically obvious cause, occult (GI) bleeding should always be considered, with studies demonstrating GI abnormalities in 62%-90% of patients [14,15].

Although, the diagnosis of anaemia is considered as important in primary care, in a changing and uncertain world where the health care budgets are increasingly revisited, the need for an inexpensive and accurate diagnostic technology is becoming promising as option. Although the use of near-patient testing (point-of-care devices) is still a controversial issue introducing opposite views between clinical practitioners and laboratory physicians it attracts the interest of academic and laboratory researchers [16]. It seems that the use of such diagnostic tools could lead to the identification of new cases of anaemia or other conditions, earlier than usually identified [17]. In Greece, this seems to be rather neglected and there is limited information that reports the burden due to anaemia, and there are questions whether many cases have been undiagnosed with anaemia among patients visiting frequently rural primary care centers. It is also worthy to discuss to what extent inexpensive diagnostic tools can contribute to early diagnosis of anaemia. Towards this direction a multisetting study on anaemia diagnosis using a simple and low cost diagnostic tool was designed in rural Crete [17].

The primary focus of this cross-sectional study was to identify new cases of anaemia among patients in regular contact with their GP in rural Crete. A secondary focus was to explore the frequency of IDA among the new cases of anaemia.

## Methods

### Setting

Between October and November 2009, all active members (n = 17) of the recently developed practice-based research network, coordinated by the Clinic of Social and Family Medicine, School of Medicine, University of Crete, were eligible and they were invited to participate in this study [18]. Fourteen GPs from nine primary health care units in Crete accepted this invitation. The total population coverage of these primary care units was estimated to be approximately 40,000 people.

### Participants recruitment and sampling

All primary care users who were visiting the participating GPs in rural Crete, over ten consecutive working days, were eligible to participate. Adult patients who volunteered to participate in the research were required to meet the following criteria: they were visiting their GPs for a routine medical reason; they were "known" to the GP (number of visits of each participating patient was at least two during the last year). Patients who were already diagnosed with anaemia, and those currently receiving treatment or who had received treatment for anaemia within the last six months were excluded from the study. Additionally, patients who had a malignancy in their medical history were excluded. The patients were fully informed by the GP regarding the procedure and objectives of the study and they were asked to give written consent for their participation. Given an expected proportion of anaemia in this population group (prevalence) of 10% (Pr = 0.1), the sample size needed, with a precision of 3% (d = 0.03) and a 95% confidence level, is at least 384 [19].

### Test methods

**Figure 1:**
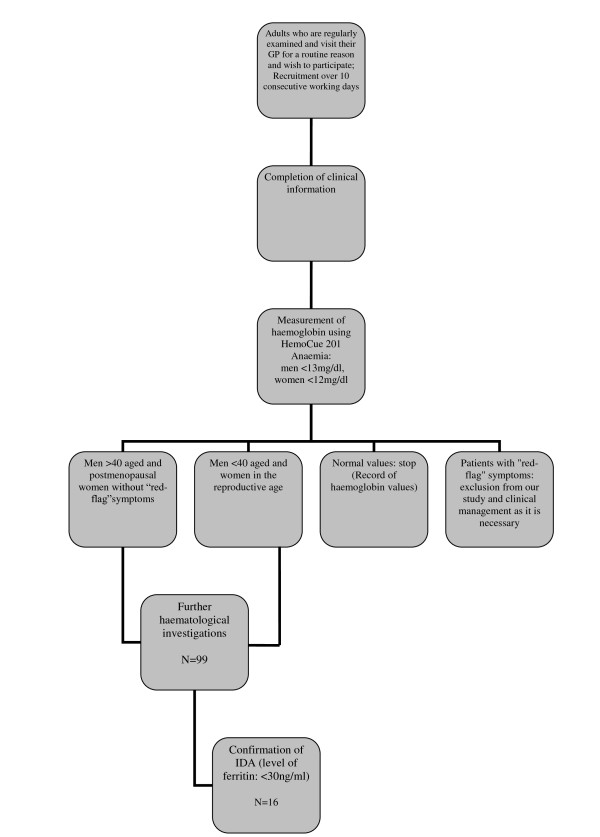
**A flowchart summarizes the protocol developed for the detection of new cases of iron deficiency anaemia**.

Blood collection was performed initially by finger prick testing [20] in the GP practices, then if required by venepuncture for laboratory investigation after providing patients with detailed information on the procedures. Hb was measured with the use of a portable device (HemoCue 201). Well informed and trained GPs executed the HemoCue test and read the results from this portable device. For the patients who had possible anaemia according to the portable device measurements, an appointment was arranged to collect blood samples on a specific date (Figure 1). Anaemia was defined when the Hb level (with the use of the portable analyzer) was < 13 g/dl in men or < 12 g/dl in women, in accordance with the WHO criteria for detecting anaemia [21]. For each of these patients, the laboratory investigations requested included full blood count and serum ferritin. The testing of all samples took place in the Laboratory of the University General Hospital of Heraklion (Beckman Coulter^® ^LH 780 Hematology Analyzer). Prior to collection of samples, the researchers took instructions regarding transport of samples from the various rural sites within a suitable timeframe, as a precaution to avoid sample degradation. IDA was diagnosed when the Hb levels were < 13 g/dl in men or < 12 g/dl in women in combination with levels of ferritin lower than 30 ng/ml [22].

### Statistical analysis

The data obtained were tabulated in a Microsoft Excel database and were then analysed with the use of SPSS 17.0 (SPSS Inc.; Chicago, IL, USA). Standard descriptive statistics, including means, standard deviations, 95% Confidence Intervals (CI) and frequency counts were used to characterize the sample. Pair t-test was also performed. A difference of P < 0.05 was considered significant.

### Ethics

This study was designed by the Clinic of Social and Family Medicine and was approved by the Commission of Bioethics of the University General Hospital of Heraklion (number of protocol: 4578, 07.05.2008). A written, fully informed consent was obtained from all adult patients enrolled into the study. All GPs and researchers involved in this study signed an agreement that the protocol and the emerging information would be strictly confidential.

## Results

### Participants

**Figure 2:**
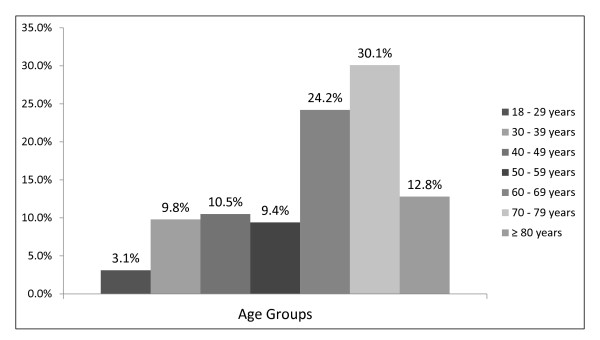
**Age of the participants**.

**Figure 3:**
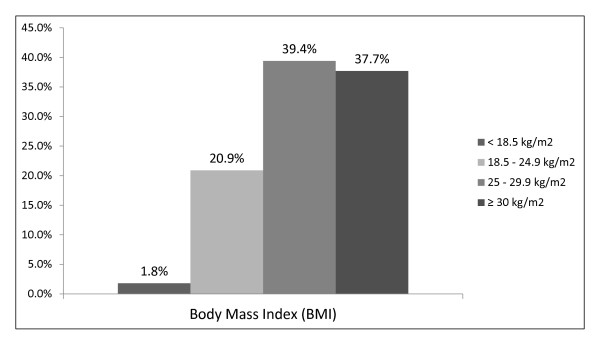
**Body Mass Index (BMI) of the participants**.

From the initial total number of GPs involved in the study, two were excluded due to practical limitations related to patient recruitment. Of the remaining twelve participating GPs, a total of 541 patient attendees were recruited, of whom 213 (39.4%) were males and 328 (60.6%) were females. The age groups represented in higher frequencies were those of 70-79 years and 60-69 years (Figure 2). The collection of somatometric data showed that mainly persons with a high Body Mass Index participated in this study (Figure 3).

### Anaemia tested at practice

The finger prick capillary sampling indicated 113 (20.9%) out of 541 study participants with possible anaemia; 45 of them were men (39.8%), and 68 were women (60.2%). Of the 113 subjects with possible anaemia, 14 patients declined to further participate and the remaining 99 patients (18.3% of the total sample) had blood tests performed in the Laboratory of the University General Hospital of Heraklion. Forty five patients (45/99, 45.5%) (male to female ratio 1:2) were confirmed with anaemia with a positive predictive value of the HemoCue test of 45.6%. The mean age of the men and women with confirmed anaemia were 77.6 (SD: 4.9) and 67.1 (SD: 17.1) respectively.

Sixteen patients, (male to female ratio 1:3) had a ferritin level lower than 30 ng/ml. The frequency of an IDA diagnosis, based on the ferritin level, was 16.2% of those who underwent laboratory testing and 3% of the initial sample. Of the patients with IDA, the mean age for males was 73.8 years (SD: 2.1) and for females was 59.2 years (SD: 21.6). The BMI of the male patients with IDA was lower in comparison to the female patients [24.5 (SD: 5.7) vs. 28.3 (SD: 7.2)].

Based on their medical history, the underlying causes among patients with IDA, were possibly related to gastric ulcer (1 case), colon polyp (1 case), rheumatoid arthritis (2 cases), menstrual cycle disorder (5 cases) and a prosthetic heart valve (1 case). An unclear explanation of IDA status for 6 cases was recorded. During this study, a new case of a male patient (85 years old) with a proximal colon cancer was identified.

### Estimates

In the group with confirmed anaemia, the mean value of the Hb levels detected by the portable device was lower than the mean value of the Hb levels obtained from the full blood count [11.1 g/dl (95% CI from 10.9 to 11.4) vs 11.4 g/dl (95% CI from 11.2 to 11.7), (*P *= 0.01)]. Among the 54 patients whose anaemic status was not confirmed by laboratory testing, the mean value of the Hb levels identified by the portable device was lower than the mean value of the Hb levels based on the full blood count [11.8 g/dl (95% CI from 11.6 to 12.0) vs 13.4 g/dl (95% CI from 13.1 to 13.4), (*P *= 0.0001)]. For the IDA patients, the mean value of the Hb levels detected by the portable device analyzer was again lower than the mean value of the Hb levels obtained from the full blood count but without meeting statistical significance [10.9 g/dl (95% CI from 10.5 to 11.3) vs 11.3 g/dl (95% CI from 10.9 to 11.7), (*P *= 0.06)] (Table 1).

**Table 1:** Mean values of the Hemoglobine of patients with a confirmed anaemia, patients with a false positive diagnosis of anaemia, and patients with a confirmed iron deficiency anaemia

	Hb value according the portable device	Hb value according full blood count	P - Value
Patients with a confirmed anaemia (n = 45)	11.1 g/dl (95% Confidence Interval (CI): 10.9-11.4)	11.4 g/dl (95% CI: 11.2-11.7)	0.01

Patients with a false positive anaemia (n = 54)	11.8 g/dl (95% CI: 11.6-12.0)	13.4 g/dl (95% CI: 13.1-13.4)	0.0001

Patients with a confirmed Iron Deficiency Anaemia (n = 16)	10.9 g/dl (95% CI: 10.5-11.3)	11.3 g/dl (95% CI: 10.9-11.7)	0.06

## Discussion

### Main findings

The main finding of this study was that it indicated 113 patients with possible anaemia, and 45 out of them were identified as new cases with anaemia. Although the primary focus of this study was not to assess the diagnostic accuracy of the HemoCue device, 45% of those identified as having anaemia by the portable device indeed had anaemia. In the cases of confirmed anaemia however, the portable device showed similar mean Hb values to those of the full blood count.

### Highlighting other findings

The 428 patients with negative readings using the portable device cannot be proven to be 'not anaemic'. Bearing in mind the technical difficulties and the high feasibility of laboratory tests the 541 patients were not able to perform all extended exams. Thus, this study did not measure the specificity and in general the diagnostic accuracy/reliability of this portable device. However the specificity of the HemoCue 201 analyzer is reported to be 94-95% in two recent studies [21,23]. Mendrone performed the analysis of Hb with the use of the HemoCue 201 device on 969 potential female donors, and detected a sensitivity of 56% and a specificity of 93.5%, with the device being able to identify all those donors with anaemia who had Hb lower than 11.0 g/dL [21]. Eekhof and Groeneveld used the HemoCue 201 device on 58 patients of two Dutch primary care units and they discovered a higher sensitivity of 81% and a similar specificity to the previous mentioned study, of 95% [23]. Future efforts are required to confirm if the returned negative cases identified with this device are truly negative and to describe its overall utility in the local settings by also introducing issues of cost analysis. Other benefits of the portable device include the limited capacity required to perform the test and immediacy of the results [24].

Taking into consideration the 16 cases of IDA from an initial group of 541, we estimate that the frequency of new cases of IDA identified on the basis of this observatory study was approximately 3%. Anaemia due to other causes is expected to be approximately 6% (29/541 cases). Thus, the portable device can help to identify subjects who may have a higher likelihood of anaemia.

One other interesting finding in our study is the high prevalence of overweight and obesity patterns observed in our sample. All patients who were included were living in rural areas of Crete. It has been suggested from a recent study that a high prevalence of increased body weight trends was showed to occur among farmers in the areas of rural Crete [25].

### Strengths and limitations

With the use of this portable device we were able to identify 45 cases of undetected anaemia. Certain limitations interfered with the interpretation of the results of this study. Firstly, as previously noted, for reasons of financial cost we were limited to perform full laboratory testing in all 541 patients involved in the study and, consequently, to measure sensitivity, specificity, negative predictive value and likehood ratios. Additionally it was not possible to discuss cut offs of better performance of the device. Another limitation is that the finger prick capillary testing was performed at different practices by different GPs, which may introduce a measurement bias. As Conway et al. suggested, Hb measurement from single skin-puncture drops can give rise to misleading results [26]. Similarly, the venepuncture took place in different practices, although this was conducted by a single researcher. The venepuncture took place within approximately 20 to 25 days from when the initial finger prick testing was performed, a delay which was impossible to be avoided given the rural setting, but which should not have introduced bias as the lifespan of a red blood cell is longer than this interval [27]. Although this is not a diagnostic accuracy study, several items of the STARD checklist such as reporting the study population, participant sampling, statistical and test methods were followed [28].

### Clinical applicability of the study results

Despite its visible limitations, this study raises some important issues around investigation and diagnosis of anaemia in rural and remote general practice settings. It is demonstrated that the use of low cost portable analysers may successfully lead to the diagnosis of new cases of anaemia among regular primary care attendees. The use of near to patient testing for anaemia with a portable device could also shorten the process of diagnosing new cases of anaemia, thus allowing earlier therapeutic intervention. The portable device provides the Greek GP with an opportunity to make a proper referral for further investigation for the confirmation of the anaemic status of a patient, when the monitor of this device shows low Hb values. The likelihood of receiving a positive confirmation for the anaemic status would also increase when the information given by the clinical examination supports the diagnosis of anaemia. Certain questions are still unanswered based on the results of this study and further research is needed.

## Conclusions

From the study sample, 45% of those identified as having anaemia by the portable device, indeed had anaemia. To determine the probability that this device correctly predicts true anaemia a study of diagnostic accuracy is needed. Another conclusion of this study is that in rural and isolated areas, anaemia is still 'invisible'. It also seems that an ordinary visit can offer the opportunity to detect new cases of anaemia. This effort seems to be a good start in a field where healthcare system capacity in Greece is challenged by the current financial restrictions and where barriers of access to health care are expected to increase.

## Competing interests

The authors declare that they have no competing interests.

## Authors' contributions

EKS and CL conceived and shaped the idea. EKS and GD prepared the first draft of the manuscript. EKS, GD, FA, SD, CK, EL, KM, CP, NS, SS, AS, AV, and TV were involved in organizing and performing the data acquisition. CL provided clinical details and technical input, revised the manuscript and performed editing and format changes throughout the manuscript. All authors read and approved the final manuscript.
